# “La Toilette”. When a doctor becomes a painter: Frederic Bazille

**DOI:** 10.1007/s40618-019-01040-7

**Published:** 2019-04-15

**Authors:** L. Obolonczyk, M. Berendt-Obolonczyk, K. Sworczak

**Affiliations:** Department of Endocrinology and Internal Medicine, Medical University of Gdansk, Debinki 7 Street, 80-952 Gdansk, Poland

**Keywords:** Frederic Bazille, Endocrinology, Painting

## Abstract

**Purpose:**

To find endocrinological disturbances in impressionism.

**Patients and methods:**

Analysis of “La Toilette” painting of Frederice Bazille.

**Results:**

We present a masterpiece work of Frederic Bazille “La Toilette” where a large goiter is visible. Short description of Bazille’s life and painting is included.

**Conclusion:**

Despite of unique painting technique, thyroid disorders are visible even in impressionism.

Jean Frederic Bazille (1841–1870) was born in Montpellier and grew up in a wealthy, middle class family. His father was a prominent wine dealer. Due to a number of father’s connections, young Frederic met an art collector Alfred Bruyas. During this closer relationship, he could admire paintings of, e.g., Delacroix and Corot. First as a spectator, later as a young artist his painting adventure slowly started.

In 1859, Bazille started medical study in Montpellier and since 1862 continued it in Paris. A contact with impressionists as Monet, Renoir and Sisley made him more painter than doctor. He was also known as a great benefactor because of his material support for his friends (especially Monet). In 1864, he finished medical study, but he never worked as a doctor. He died at age of 29 years in Franco-German war [1].

“La toillete” oil on canvas was finished in 1870 just before Bazille’s death (Fig. 1). It presents a French art model Lise Trehot, but for us more interesting is a mysterious woman on the right side. We see clearly large, smooth goiter. No eye signs, but slim woman’s stature does not help with differentiation between simple goiter and Graves’ disease. Historically, goiter seems to be “older” disease (i.e., paintings of Flemish or Italian Renaissance painters) but this question will be unanswered [2, 3].

**Fig. 1:**
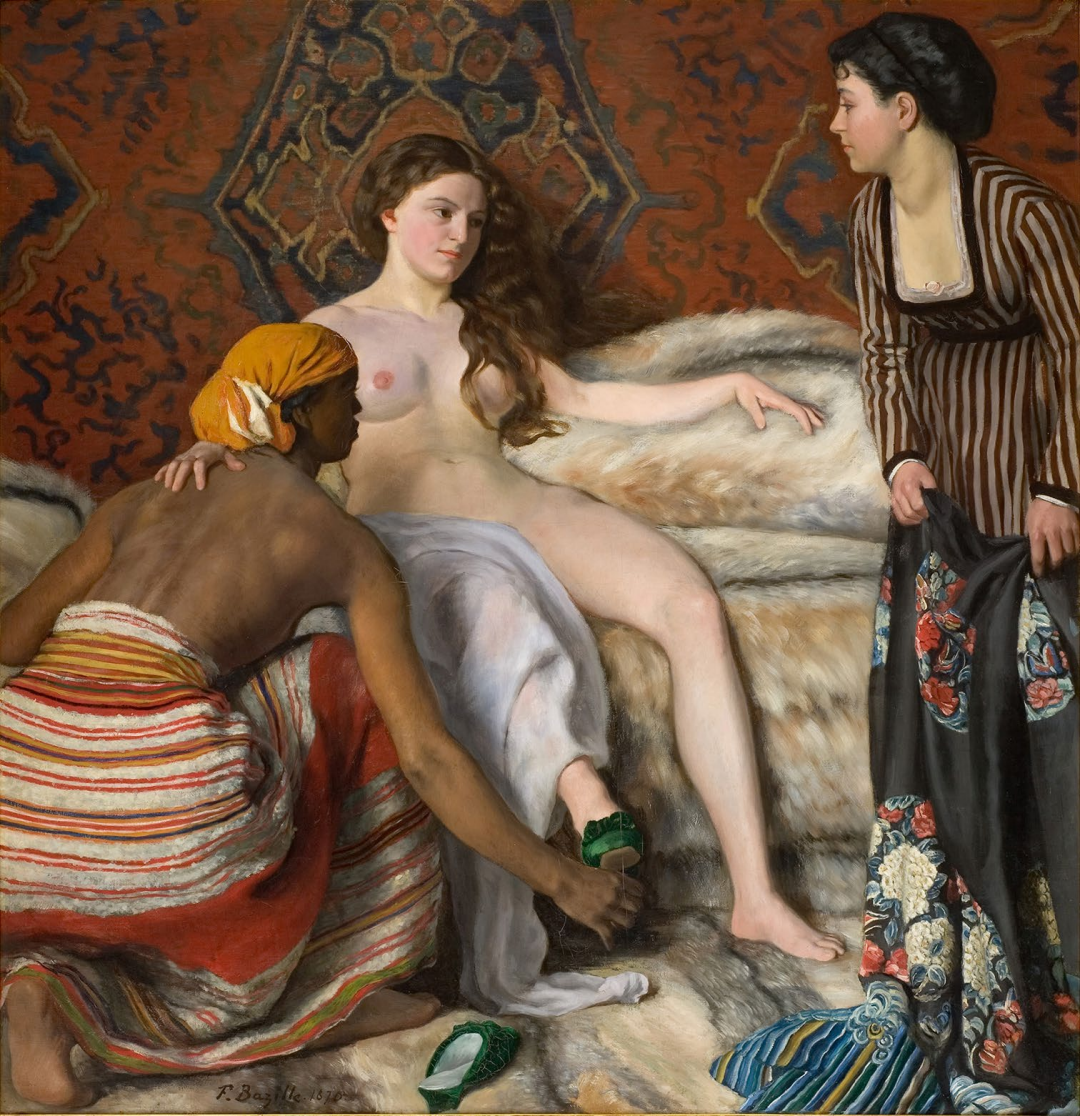
La Toilette (December 1869–March 1870), by Frederic Bazille, oil on canvas 153 × 148.5 cm [Musee Fabre, Montpellier, France]. Please note large goiter on first right lady

According to Encyclopaedia Brittanica, Bazille was an *unenthusiastic* medical student. I strongly deny this opinion when I see such perfect thyroid. I hope every student have Bazille’s perception [4].
